# Pulmonary function and factors associated with current smoking among the hill tribe populations in northern Thailand: a cross-sectional study

**DOI:** 10.1186/s12889-020-09857-1

**Published:** 2020-11-16

**Authors:** Anongnad Mee-inta, Ratipark Tamornpark, Fartima Yeemard, Panupong Upala, Tawatchai Apidechkul

**Affiliations:** 1 Department of Physical Therapy, School of Integrative Medicine, Mae Fah Luang University, Chiang Rai, Thailand; 2 Department of Public Health, School of Health Science, Mae Fah Luang University, Chiang Rai, Thailand; 3 Center of Excellence for The Hill tribe Health Research, Mae Fah Luang University, Chiang Rai, Thailand

**Keywords:** Pulmonary function, Hill tribe, Smoking behaviors, Factors associated

## Abstract

**Background:**

Smoking is considered one of the major poor health behaviors leading to several health problems. Individuals with a poor education and economic status are vulnerable to smoking, particularly the hill tribe people in Thailand. This study aimed to estimate the prevalence of current smoking, assess pulmonary function, and identify factors associated with current smoking among individuals aged 20 years and older of the hill tribes in northern Thailand.

**Method:**

A cross-sectional study was conducted to gather information from the hill tribe people living in 42 hill tribe villages. A validated questionnaire, spirometry, and pulse oximetry were used as the research tools. Face-to-face interviews were conducted to collect data from the participants in a private room after obtaining informed consent voluntarily. Chi-squared test and logistic regression were used to detect the associations between the variables at the significance level of α = 0.05.

**Results:**

In total, 2216 participants were recruited into the study: 54.6% were women, 80.3% were aged 31–59 years, and 86.1% were married. The prevalence of smoking was 36.3%; 20.6% were current smokers (36.7% among men and 7.0% among women), and 15.7% were ever smokers. Half of the participants (50.1%) had smoked for ≤9 years, 80.1% smoked ≤10 pieces per day, 64.2% smoked traditional tobacco, 42.8% had low-to-moderate levels of knowledge of the harms of smoking, and 68.4% had low-to-moderate levels of attitudes toward the harms of smoking. Only sex was statistically significant among the different smoking behaviors (*p*-value< 0.001), and the participants’ pulmonary function was not significantly different. After controlling for age, sex, religion, and education, three variables were found to be associated with current smoking among the hill tribe people in Thailand: men were more likely to smoke than women (AOR = 7.52, 95% CI = 5.53–10.24); those who used amphetamines were more likely to smoke than those who did not (AOR = 2.92, 95% CI = 1.69–5.03); those who had poor attitudes toward the harms of smoking were more likely to smoke than those who had a positive attitude toward the harms of smoking (AOR = 2.47, 95% CI = 1.53–3.97).

**Conclusion:**

Translating essential health messages into the hill tribe language and improving the channel to deliver this information to the target populations, particularly men, are crucial strategies for improving their knowledge and attitudes toward the harms of smoking and making them quit smoking.

**Supplementary Information:**

The online version contains supplementary material available at 10.1186/s12889-020-09857-1.

## Background

Smoking is an important health behavior that may lead to several health complications, such as cancer (CA) [1, 2], cardiovascular disease (CVD) [3], and chronic obstructive pulmonary disease (COPD) [4]. The diseases that develop from smoking behavior require costly care and treatment [5]. Most smokers end their lives with poor physical and mental health [6]. The impacts are not limited to individuals who smoke; they extend to their family members, community and nation through socioeconomic systems [6]. The World Health Organization (WHO) estimates that 1.4 trillion dollars are lost due to smoking among the global population [7]. The WHO reported that more than 8 million people are killed by smoking behavior each year; 87.5% are directly killed by smoking, and the others are killed by second-hand smoking [6]. A significant point is that more than 80.0% of those who die because of smoking live in developing countries, including Thailand.

Thailand is classified as a developing country [8] with an estimated total population of 67 million in 2019 [9]. The Thai Health Promotion Foundation reported that 553,611 patients were treated for issues related to smoking in 2017, with an expenditure of approximately 21,389 million baht [10]. The Ministry of Public Health Thailand estimated that, in the Thai population aged 15 years and older, 10.7 million people (19.1% prevalence) were defined as smokers in 2019, and those aged 25–44 years constituted the greatest proportion of smokers [11]. In 2018, WHO reported 114,199 deaths from cancers, and 170,495 cases of cancers were treated in Thailand [12]. Among the on-treatment cancer patients, most had lung cancer (14.1%), liver cancer (13.7%), and breast cancer (11.4%) [12]. Importantly, among smokers, the reduction in pulmonary function is an urgent issue, particularly among those working in agricultural sections who require extensive physical energy to complete their daily duties. A comparative study conducted in Thailand clearly indicated that those who smoked had a significantly reduced pulmonary function compared with those who did not [13]. The Ministry of Public Health stated that the populations most vulnerable to smoking have a poor education and economic status, such as the hill tribe people [14].

The hill tribe migrated from southern China over centuries to settle in the mountainous areas of northern Thailand, far from large cities [15]. The WHO reported that six main groups of hill tribes exist in Thailand—Akha, Lahu, Hmong, Yao, Karen, and Lisu—comprising approximately 3.5–4.0 million people in 2017 [16]. All hill tribe people have their lifestyles and patterns of culture, with some patterns related to substance use behaviors, including smoking [17]. Given their living conditions, particularly poor economic conditions and poor education, the hill tribe people are highly likely to adopt smoking behaviors. Apidechkul et al. [18] reported that more than 30.0% of hill tribe people were not granted the Thai identification card (ID), which is used to access public services, including free medical services [19]. Therefore, for people who have health problems, access to medical services is difficult.

There is very limited scientific information on smoking behaviors and pulmonary function. This study aimed to estimate the prevalence of smoking, assess pulmonary function, and identify the factors associated with current smoking behavior among people of the hill tribe aged 20 years and older in northern Thailand.

## Methods

### Study design

A cross-sectional study design was used to gather information from the participants.

### Study population

The study population was the hill tribe people aged 20 years and older. The targeted hill tribe population in the study was one of the six main tribes: Akah, Lahu, Hmong, Yao, Karen, and Lisu. Hill tribe people living in 42 selected hill tribe villages in Chiang Rai Province, Thailand, were eligible for the study. Those who were unable to provide essential information regarding the study protocols were excluded from the study.

### Study sample

The sample size was calculated based on the standard formula of a cross-sectional design [20], where Z^2^_α/2_ = 1.96, *P* = 0.41 [21], Q = 0.59, and e = 0.05. Therefore, 360 participants were required from each tribe. In total, 6 tribes and 2162 participants were needed for the analysis.

### Research instruments and their development

Three research instruments were used to collect data: validated questionnaires, spirometry, and pulse oximetry. A questionnaire was developed based on a review of the literature and information obtained from discussion with health professionals working in small health centers located in the hill tribe villages. The questionnaire was divided into five parts. In part one, 11 questions were used to collect general information about the participants, such as age, sex, education, and marital status. In part two, 15 questions were used to collect information on smoking behaviors, such as type of smoking, duration of smoking, sources of obtaining cigarettes, and money used to buy cigarettes per day. In part three, 5 questions were used to collect information on substance use, such as alcohol use behavior, amphetamine use, and opium use. In part four, ten questions were used to detect knowledge of the harms of smoking, and another ten questions were used to collect data on attitudes toward the harms of smoking. In part five, four open-ended questions were used to obtain information on pulmonary function testing with forced expiratory volume in the first second (FEV_1_), the volume delivered during an expiration made as forcefully and completely as possible starting from full inspiration (FVC), the ratio of FEV_1_ and FVC (FEV_1_/FVC), and O_2_ saturation.

The validity of the questionnaire was tested by asking three external experts who were working in the field—one epidemiologist, one physical therapist, and one public health professional—for the item-objective congruence index (IOC). Subsequently, a pilot test was conducted at Mae Fah Luang District comprising 20 participants with similar characteristics to the study population. In the pilot test, the same sample was assessed three times to test the feasibility, ordering of the questions, and proper questions for the hill tribe. The Cronbach’s alpha of the sections on knowledge and attitudes were 0.76 and 0.71, respectively (Additional file 1, Questionnaire).

To assess pulmonary function, a standardized pulmonary function test of the American Thoracic Society (ATS) [22] was used. Normal was classified as FEV_1_ ≥ 80.0%, FEV_1_/FVC ≥70.0, and 94.0%–100.0% oxygen saturation.

### Process of data gathering

The hill tribe villages were selected by a random method from lists of the 6 tribe villages located in Chiang Rai Province. Based on the information in 2018, 652 hill tribe villages, including 243 Akha villages, 216 Lahu villages, 63 Yao villages, 59 Hmong villages, 36 Karen villages, and 35 Lisu villages, were identified [23]. Given the different numbers of village members, 42 hill tribe villages were selected for the study: 5 Akha villages, 8 Lahu villages, 7 Hmong villages, 6 Yao villages, 8 Karen villages, and 8 Lisu villages. Access to the selected villages was granted by the district government officers. The target village headmen were contacted and asked for a list of people who met the criteria. An appointment was made five days before collecting the data. On the day of data collection, all the participants were informed of all essential information and asked to provide informed consent before starting the interview. Each interview and pulmonary function assessment lasted 30 min.

### Statistical analysis

The questionnaires were coded and double-entered into SPSS version 24 (SPSS, Chicago, IL). Descriptive statistics were used to explain the general characteristics of the participants. Continuous data are described as means and SD, while categorical data were described as percentages. Chi-squared test was used to detect the difference in proportions between variables. Logistic regression was used to detect the associations of independent variables and current smoking at the significance level of α = 0.05. In the current study, smokers were coded as “1”, while nonsmokers and never smokers were coded as “0”. “Enter” was used for both univariate and multivariate analyses. The pseudo R^2^ of the Cox-Snell R^2^ and Nagelkerke’s R^2^ were used to determine the fit of the model in all steps. In the final step, age and sex were controlled as confounding factors in the model before interpretation.

## Results

In total, 2216 participants were recruited into the study; 54.6% were women, 80.3% were aged 30–59 years, and 86.1% were married. The average age of the men was 49.7 years (SD = 11.3) and that of the women was 46.8 years (SD = 10.1), with a statistically significant difference (*p*-value< 0.001). The proportions of participants by tribes were 14.8–19.0%. More than half were Buddhist (54.8%), uneducated (53.4%), and had annual family income of 10,000–50,000 baht (59.6%) (Table 1).

**Table 1:** General characteristics of the participants

Characteristics	***n***	%
**Total**	2216	100.0
**Sex**		
Male	1007	45.4
Female	1209	54.6
**Age** (years)		
20–29	41	1.9
30–39	279	20.7
40–49	717	32.4
50–59	602	27.2
≥ 60	398	18.0
Mean = 48, S.D. = 10.8, Min = 25, Max = 78		
**Tribe**		
Akha	421	19.0
Lahu	387	17.5
Hmong	364	16.4
Yao	346	15.6
Karen	329	14.8
Lishu	369	16.7
**Marital status**		
Single	126	5.7
Married	1908	86.1
Widowed	111	5.0
Divorce	71	3.2
**Religion**		
Buddhism	1214	54.8
Christian	1002	45.2
**Education**		
Uneducated	1184	53.4
Primary education	573	25.9
Secondary education	341	15.4
High vocational	83	3.7
University	35	1.6
**Annual family income** (Baht)		
≤ 10,000	210	9.5
10,001-50,000	1321	59.6
50,001-100,000	466	21.0
≥ 100,001	213	9.9
**Occupation**		
Unemployed	186	8.4
Merchant	53	2.4
Employee	233	10.5
Agriculture	1744	78.7

Regarding smoking behaviors, 36.3% were currently smoking, 50.1% smoked for 9 years or less, 80.1% smoked 10 pieces or less per day, 64.2% used traditional tobacco, and 89.6% spent less than 50 baht per day (2$US). Less than half (42.8%) had low to moderate levels of knowledge of the harms of smoking, and 68.4% had low to moderate levels of attitudes toward the harms of smoking. Regarding other substances, 42.5% used alcohol, 4.5% used amphetamines, and 4.2% used opium (Table 2).

**Table 2:** Characteristics of smoking and substance use behaviors

Characteristics	***n***	%
**Smoking**		
No	1412	63.7
Ever	349	15.7
Yes	455	20.6
**Length of smoking** (Years)		
≤ 9	403	50.1
10–19	252	31.3
≥ 20	149	18.6
Mean = 15, SD = 10.6, Min = 1, Max = 53		
**Amount of smoking** (Cigarettes/day)		
≤ 10	644	80.1
11–19	61	7.6
≥ 20	99	12.3
Mean = 8, SD = 6.78, Min = 1, Max = 50		
**Types of cigarette**		
Traditional tobacco	516	64.2
Commercial cigarette	285	35.4
Electronic cigarette	3	0.4
**Smoking in family**		
Yes	238	29.6
No	566	70.4
**Source of cigarette buying**		
From their village	751	93.4
Outside of their village	53	6.6
**Cost of cigarettes per day** (Baht)		
Less than 50 baht	720	89.6
More than 50 baht	84	10.4
**Smoking in house** (Indoor building)		
Yes	576	71.6
No	228	28.4
**Knowledge of the harms of smoking**		
Low	219	9.9
Moderate	730	32.9
Good	820	37.0
**Attitudes toward the harms of smoking**		
Low	716	32.3
Moderate	801	36.1
Good	252	11.4
**Alcohol use**		
Yes	942	42.5
No	1274	57.5
**Amphetamine use**		
Yes	100	4.5
No	2116	95.5
**Opium use**		
Yes	92	4.2
No	2124	95.8
**Marijuana use**		
Yes	59	2.7
No	2157	97.3

Comparing smoking behaviors and pulmonary function, only sex was found to be statistically significant (*p*-value< 0.001). Pulmonary function was not significantly different among the different groups of smoking behaviors (Table 3).

**Table 3:** Comparison of the spirometry outcomes by sex among people with smoking experience

Characteristics	Smoking behavior						χ^**2**^	***p***-value
	Yes		Ever		No			
	***n***	%	***n***	%	***n***	%		
**Sex**								
Male	370	36.7	284	28.2	353	35.1	655.97	< 0.001*
Female	85	7.0	65	5.4	1059	87.6		
**FEV**_**1**_ **(%)**								
Normal (≥80)	262	22.6	207	17.9	689	59.5	0.24	0.884
Low (< 80)	136	23.7	101	17.6	337	58.7		
**FVC (%)**								
Normal (≥80)	187	23.7	130	16.5	473	59.9	1.84	0.398
Low (< 80)	211	22.4	178	18.9	553	58.7		
**FEV/FVC**								
Normal (≥0.8)	397	23.1	305	17.7	1017	59.2	1.74	0.460^a^
Low (< 0.8)	1	7.7	3	23.1	9	69.2		
**Oxygen saturation (%)**								
Normal (94–100)	443	20.4	339	15.6	1388	64.0	2.75	0.252
Lower (< 94)	12	26.1	10	21.7	24	52.2		

* Significant difference at α = 0.05, ^a^Fisher’s exact test

In univariate analyses, 12 variables were associated with current smoking: sex, age, tribe, marital status, religion, education, occupation, alcohol use, amphetamine use, opium use, marijuana use, and attitudes toward the harms of smoking.

In multivariate analysis, after controlling for age, sex, religion, and education, three variables were found to be associated with current smoking among the hill tribe people in Thailand. Men were 7.52 times more likely to smoke than women (95% CI = 5.53–10.24). Those who used amphetamines were 2.92 times more likely to smoke than those who did not (95% CI = 1.69–5.03), and those who had low attitudes toward the harms of smoking were 2.47 times more likely to smoke than those who had a good attitude toward the harms of smoking 2 (95% CI = 1.53–3.97) (Table 4).

**Table 4:** Univariate and multivariate analyses to identify factors associated with current smoking

Characteristics	Current smoking				OR	95% CI	***p***-value	AOR	95% CI	***p***-value
	Yes		No							
	***n***	%	***n***	%						
**Sex**										
Male	370	36.7	637	63.3	7.68	5.95–9.91	< 0.001*	7.52	5.53–10.24	< 0.001*
Female	85	7.0	1124	93.0	1.00			1.00		
**Age** (years)										
20–29	14	34.1	27	65.9	1.99	1.00–3.98	0.049*			
30–39	78	17.0	380	83.0	0.79	0.56–1.11	0.182			
40–49	148	20.6	569	79.4	1.00	0.74–1.35	0.988			
50–59	133	22.1	469	77.9	1.09	0.80–1.49	0.575			
≥ 60	82	20.6	316	79.4	1.00					
**Tribe**										
Akha	95	22.6	326	77.4	1.00					
Lahu	89	23.0	298	77.0	1.02	0.73–1.42	0.884			
Hmong	23	6.3	341	93.7	0.23	0.14–0.37	< 0.001*			
Yao	92	26.6	254	73.4	1.24	0.89–1.72	0.197			
Karen	90	27.4	239	72.6	1.29	0.92–1.80	0.131			
Lishu	66	17.9	303	82.1	0.74	0.52–1.06	0.104			
**Marital status**										
Single	43	34.1	83	65.9	1.00					
Married	378	19.8	1530	80.2	0.47	0.32–0.70	< 0.001*			
Widowed	19	17.1	92	82.9	0.39	0.21–0.73	0.003*			
Divorce	15	21.1	56	78.9	0.51	0.26–1.01	0.057			
**Religion**										
Buddhist	185	15.9	982	84.1	1.00					
Christian	270	26.9	732	73.1	1.95	1.58–2.41	< 0.001*			
**Education**										
Uneducated	222	18.8	962	81.3	1.00					
Primary education	155	27.1	417	72.9	1.60	1.27–2.03	< 0.001*			
Secondary education	56	16.4	285	83.6	0.85	0.61–1.17	0.327			
High Vocational Certificate	11	13.3	72	86.7	0.66	0.34–1.26	0.214			
Bachelor’s degree or higher	11	31.4	24	68.6	1.98	0.95–4.11	0.065			
**Annual family income** (baht)										
≤ 10,000	36	17.1	174	82.9	1.00					
10,001-50,000	306	23.2	1015	76.8	1.45	0.99–2.13	0.053			
50,001-100,000	80	17.2	386	82.8	1.00	0.65–1.54	0.994			
≥ 100,001	33	15.1	186	84.9	0.85	0.51–1.43	0.559			
**Occupation**										
Unemployed	28	15.1	157	84.9	1.00					
Merchant	5	9.4	48	90.6	0.58	0.21–1.60	0.300			
Employee	71	30.5	162	69.5	2.47	1.51–4.03	< 0.001*			
Agriculture	351	20.1	1393	79.9	1.42	0.93–2.16	0.099			
**Alcohol use**										
Yes	278	29.5	664	70.5	2.59	2.10–3.20	< 0.001*			
No	177	13.9	1097	86.1	1.00					
**Amphetamine use**										
Yes	49	49.0	51	51.0	4.04	2.69–6.07	< 0.001*	2.92	1.69–5.03	< 0.001*
No	406	19.2	1710	80.8	1.00			1.00		
**Opium use**										
Yes	35	38.0	57	62.0	2.49	1.61–3.84	< 0.001*			
No	420	19.8	1704	80.2	1.00					
**Marijuana use**										
Yes	25	42.4	34	57.6	2.95	1.74–5.00	< 0.001*			
No	430	19.9	1727	80.1	1.00					
**Knowledge of the harms of smoking**										
Low	45	20.5	174	79.5	1.00	0.69–1.45	0.984			
Moderate	131	17.9	599	82.1	0.84	0.65–1.09	0.849			
Good	168	20.5	652	79.5	1.00					
**Attitudes toward the harms of smoking**										
Low	195	27.2	521	72.8	3.25	2.10–5.04	< 0.001*	2.47	1.53–3.97	< 0.001*
Moderate	123	15.4	678	84.6	1.57	1.00–2.47	0.047*	1.36	0.84–2.21	0.203
Good	26	10.3	226	89.7	1.00			1.00		
**FEV**_**1**_ **(%)**										
Normal	262	22.6	896	77.4	1.00					
Low	136	23.7	438	76.3	1.06	0.83–1.34	0.619			
**FVC (%)**										
Normal	187	23.7	603	76.3	1.00					
Low	211	22.4	731	77.6	0.93	0.74–1.16	0.531			
**FEV**_**1**_**/FVC**										
Normal	397	23.1	1322	76.9	1.00					
Low	1	7.7	12	92.3	0.21	0.36–2.14	0.219			
**Oxygen saturation (%)**										
Normal	443	20.4	1727	79.6	1.00					
Low	12	26.1	34	73.9	1.37	0.70–2.67	0.348			

*Significant difference at α = 0.05, after adjusting for potential confounders (sex, age, education, and religion)

## Discussion

In this study, the hill tribe people aged 20 years and older living in Thailand had a poor education and economic status. One-third were smokers (36.3%) and spent a considerable amount of money buying cigarettes, even with a low family income. They used different substances but at a low rate. Pulmonary function and oxygen saturation did not differ among the nonsmokers, ever smokers, and current smokers. Differences were found among current smokers regarding sex, tribe and age group. Characteristics that served as influencing factors of current smoking were sex, amphetamine use, and attitudes toward the harms of smoking.

The Ministry of Education, Thailand reported that 96.0% of the Thai population aged 15–59 years were educated under the Thailand educational system; 22.0% earned a university degree, 20.0% graduated at high school level, 19.0% graduated at the secondary school level, and 35.0% graduated at the primary school level [24]. However, among the hill tribe people, 53.4% never attended any educational system in Thailand. Concerning family income, the National Statistical Office, Thailand reported that the average annual family income of the Thai population was 323,352 baht (10,778US$) while it was 42,000 baht (1400US$) among the hill tribe family per year [25]. The information could reflect that the hill tribe people in Thailand have lower education and income levels than the Thai general population.

Our study found that the prevalence of current smoking among hill tribe people aged 20 years and older was 20.5%, and men (36.7%) constituted a greater proportion of smokers than women (7.0%). However, no differences were found in the capacities (FEV_1_, FVC, FEV_1_/FEC) and oxygen saturation of the pulmonary system among ever smokers, current smokers, and nonsmokers. The prevalence was similar to that in a nationwide study in Poland in 2019 of 1011 participants aged 15 years and older [26], at 21.0%. The prevalence among European students was 66.1%, and the mean age at the initiation of smoking was 16 years [27]. Studies from different countries have reported different prevalence rates of smoking: 54.0% in China [28], 67.8% in Japan [29], and 22.8% in Malaysia [30]. In the data analysis of different tobacco control interventions in Thailand [31], the prevalence was 29.2% in Thailand, slightly greater than the prevalence among the hill tribe people. Gautami et al. [32] reported that the prevalence of smoking among the female Thai population aged 15 years and older was 3.4%, and people living in rural northern regions reported the highest prevalence, which was lower than that of hill tribe women.

Interestingly, pulmonary function was not significantly different among those who currently smoked, ever smoked, and never smoked. A study conducted in the United States reported that former smokers and current smokers had a significantly poorer lung function than never smokers [33]. A study in Pakistani youths in 2018 reported that smoking behaviors were associated with declining pulmonary function [34]. In Thailand, a community-based cross-sectional study reported that long-term smoking behaviors affected potential lung function [13]. To detect the association between smoking behaviors and lung function, a stronger study design, such as a case-control or a cohort study, is needed.

In this study, we found that men had a 7.52 times greater chance of being a current smoker than women among hill tribe people aged 20 years and older. This finding coincides with a study in Ethiopia [35] reporting that men had a significantly greater chance of being current smokers than women. The finding was also supported by a study in Japan, reporting that men were at a greater risk of being current smokers than women among Japanese adults [36]. Additionally, a multifactor study in Korea reported that men had a greater likelihood of current smoking than women [37]. Moreover, men living in northern Thailand had a statistically greater risk of current smoking than women [38]. A triple country study in 2019 reported that male sex was a strong factor associated with current smoking in the Thai population [39].

A study in the United States in 2017 reported that the use of some substances, such as amphetamines, led to cigarette use [40]. Chomchoei et al. [41] reported that smoking could lead to the initiation of amphetamine use, which could also lead to smoking in the Akha and Lahu adult populations in northern Thailand. A qualitative study in Thailand reported that cigarette use and amphetamine use were significant among the young Thai population [42].

Among the hill tribe adults aged 20 years and older, those with poor attitudes toward the harms of smoking had greater odds of current smoking than those with good attitudes toward the harms of smoking. This finding coincides with that in a study in China [43] reporting that those with a better attitude toward the harms of smoking had less opportunity to smoke and a greater chance to quit smoking than those with a poor attitude. Additionally, a study in Taiwan among military conscripts [44] found that those with a better attitude toward the harms of smoking were at a lower risk of current smoking than those with poor attitudes toward the harms of smoking. A study of nursing students in Italy [45] demonstrated that having a good attitude toward the harms of smoking was associated with a lower risk of current smoking than those with a poor attitude toward the harms of smoking. A cross-sectional study in Lebanon [46] also reported that having a good attitude toward the harms of smoking was protective against current smoking behaviors among university students. This finding was similar to that in a study among medical students in Argentina [47] reporting that having a good attitude toward the harms of smoking was a protective factor against current smoking behaviors. A study in Bangkok [48], central Thailand, reported that poor knowledge of the harmful effects of smoking was a risk factor for current smoking.

Some limitations were found during this study. First, the ability of some participants to completely understand the content of the study was limited because their knowledge of Thai was limited. However, the village health volunteers were asked to help explain some questions on the questionnaire before completion of the form. Second, during the collection of information on pulmonary function using spirometry, the participants needed to strictly follow the instructions to ensure that the correct results were obtained. However, elderly persons did not clearly understand or correctly follow the instructions. This issue was improved by explanations of the details of the instructions by local health volunteers who were fluent in Thai and their local languages.

## Conclusion

The hill tribe people in northern Thailand live with a poor education and economic status. Moreover, hill tribe people aged 20 years and older face a high prevalence of smoking behaviors and the use of several substances. Most of them smoke traditional tobacco and easily obtain cigarettes in their villages. Smoking behaviors are dominant in men, those with a poor attitude on smoking harms, and among those who used amphetamine. The hill tribe people must improve their knowledge on the harms of smoking to improve their attitude eventually. This point is critical for reducing smoking behavior, particularly in the male population. However, to improve knowledge, public health professionals must simplify the method and tools for providing health education because more than half of the hill tribe people are not educated. Moreover, all health messages should be translated in the hill tribe languages to achieve ultimately successful communication.

## Supplementary information

Additional file 1. Questionnaire.

## Data Availability

The raw data are available upon reasonable request from the corresponding author.

## Abbreviations

ATS: American Thoracic Society
CA: Cancer
CI: Confidence interval
COPD: Chronic obstructive pulmonary disease
CVDs: Cardiovascular diseases
ID: Identification card
IOC: Item objective congruence index
FEV_1_: Forced expiratory volume in the first second
FVC: Forced vital capacity
FEV_1_/FVC: Ratio of FEV_1_ and FVC
O_2_: Oxygen
SD: Standard deviation
WHO: World Health Organization
