# Supplementary material for: Pulmonary function and factors associated with current smoking among the hill tribe populations in northern Thailand: a cross-sectional study
Source: BMC Public Health. 2020 Nov 16;20:1725. doi: 10.1186/s12889-020-09857-1 (PMC7670626; doi:10.1186/s12889-020-09857-1)
Supplement: Supplementary file 1 — Questionnaire. [file 12889_2020_9857_MOESM1_ESM.pdf]

Questionnaire for assessing among behaviors and punmonar capacity among the hill tribe people

Weight ....kg. Height ....cm. SBP (1) .... mmHg

DBP (2) .... mmHg Heart Rate.......................... beat/min

Part 1 Demographic data

1.1 Gender ☐ male ☐ female

1.2 Age …………………. years

1.3 Did you have the Thai ID card? ☐ Yes ☐ No

1.4 Tribe

☐ Akah ☐ Lahu ☐ Hmong ☐ Yao ☐ Lisu ☐ Karen

1.5 Marital status

☐ Single ☐ Married ☐ Widow ☐ Divorce

1.6 Religion

☐ Buddhism ☐ Christian ☐ Muslim

1.7 Education

☐ Uneducated ☐ Primary education ☐ Secondary education

☐ High vocational ☐ University

1.8 Annual family Income (bath)

☐ <10,000 bath ☐ 10,001 - 50,000 bath ☐ 50,001 - 100,000 bath

☐ >100,001 bath

1.9 Number of family members ....

1.10 Right to access medical services

☐ No ☐ Universal coverage scheme ☐ Government or State Enterprise Officer

☐ Social Security Scheme ☐ Private Health Insurance

1.11 Occupation

☐ Unemployed ☐ Merchant ☐ Employee ☐ Agriculturalist

Part 2 Smoking behavior

2.1 Have you ever smoked?

☐ No

☐ Ever, I had been smoked for ……….. years and now quitted for ……….. years

☐ Yes, I am smoker and have smoked for …………… years

2.2 How often have you smoked?

☐ Everyday ……… piece/day ☐ Someday with ……… piece/day ☐ Seldom

2.3 What is the best category you are currently?

☐ Non-smoker ☐ Quitted smokers ☐ Socialized smoking ☐ Current smokers ☐ Not sure

2.4 How old were you when you first smoked a cigarette?

2.5 Please provide the reasons of smoking

☐ For relaxation ☐ Persuade by friends ☐ Eager to do ☐ Imitate the movie ☐ Follow the family member ☐ From the advertisement ☐ Smoke during drinking ☐ From an amusement centre ☐ Fashionable

2.6 Which types of cigarette do you smoke?

☐ Commercial cigarette ☐ Traditional tobacco ☐ Electronic cigarette ☐ Baragu ☐ Other ………………

2.7 When was the recent time you had smoked a cigarette?

☐Today ☐ Yesterday ☐ 2-7 days prior ☐ One 1 week to 1 month prior ☐ 1-6 months ago ☐ > 6 months to 1 year ago ☐ > 1 year ago

2.8 Last week, how many piece of cigarette do you smoke per day?

☐ Not smoke ☐ < 1 piece ☐ piece/day ☐ 2-5 pieces/day ☐ 6-10 pieces/day ☐ 11 –20 pieces/day ☐ >20 pieces/day

2.9 Do you have a family member smoked?

☐ No

☐ Yes (☐ Father ☐ Mother ☐ Brother/sister ☐ Younger sister/brother ☐ Uncle ☐ Aunt)

2.10 Do you have friends who smoke smoking?

☐ No ☐ Yes, …. person (s)

2.11 Where you often buy a cigarette?

☐ Family mart ☐ Village grocery ☐ Outside of their village

2.12 Have you ever though to quite smoking?

☐ Yes ☐ No

2.13 How much do you want to quit smoking?

☐ No, I did not ☐Rarely ☐ Yes

2.14 Have you even been quit smoking?

☐ Yes ☐ No

2.15 How do you plan to quit smoking ?

☐ I plan to quit smoking in the next 1 month

☐ I plan to quit smoking in the next 6 months

☐ I have no plan quit smoking.

☐ I have already quitted smoking.

2.16 Do you feel bad on having smoking?

☐ Very much ☐ Little ☐ Never

2.17 How much money do you use for buying a cigarette per day?…………baht

2.18 Within the month, have you use your own money to buy a cigarette?

☐ No ☐ Yes

2.19 How difficult to get a cigarette when you need it?

☐ Very difficult ☐ Little ☐ Easy

2.20 Have you ever smoked in indoor?

☐ Yes ☐ No

2.21 Within the last 7 days, how often of your family members had smoked indoor?

☐ No ☐ 1-2 times ☐ 3-6 times ☐ Everyday

2.22 If anyone starts to smoke, is it difficult to quite in your idea?

☐ Difficult ☐ Some difficult ☐ Easy

2.23 During 30 days prior, how often did you observed the warning labeled on the cigarette packages?

☐ No. ☐ Sometimes ☐ Very often

2.24 Regarding the warning labeled on the cigarette package, how often do you concern on the harms?

☐ No ☐ Little ☐ Yes

2.25 Cigarette package labeled, is it valid in your idea?

☐ No ☐ Some ☐ Yes

2.26 Did you think the warning labeled on cigarette packages, it is persuaded the same age of you do not to start use it?

☐ No ☐ Little ☐ Yes

2.27 Have you had a family member who aged 0-6 years?

☐ Yes ☐ No

2.28 Have you had a family member who aged 7-15 years?

☐ Yes ☐ No

2.29 Have you had a family member who aged 60 years and over ?

☐ Yes ☐ No

2.30 Have you had a family member who were diagnosed as lung diseases or asthma?

☐ Yes ☐ No

2.31 Have you had a family member who were diagnosed as the cancer?

☐ Yes ☐ No

Part 3 Substances use behaviors

3.1 Have you ever drank alcohol?

☐ Yes, it takes for............year ☐ Ever, it took for………....years ☐ No

3.2 Have you ever took amphetamine?

☐ Yes, it takes for............year ☐ Ever, it took for………....years ☐ No

3.3 Have you ever took heroine?

☐ Yes, it takes for............year ☐ Ever, it took for………....years ☐ No

3.4 Have you ever used an opium?

☐ Yes, it takes for............year ☐ Ever, it took for………....years ☐ No

3.5 Have you ever used a marijuana?

☐ Yes, it takes for............year ☐ Ever, it took for………....years ☐ No

Part 4.Knowledge and attitude according to harms of smoking

4.1 Knowledge according to harms of smoking

**Items**	**Correct**	**Incorrect**	**Not sure**
1. Smoking causes the lung disease.			
2. Smoking experience causes the tobacco addiction in later			
3. Filtered tobacco can reduce the harms than of non-filtered cigarette.			
4. Smoking during pregnancy is not risk of the abortion.			
5. Smoking effects health of the others.			
6. Asthmatic or allergic patients can be easily exacerbated by the cigarette smoke.			
7. Smoking effects to the brain function.			
8. Regular smoking can develop lung cancer.			
9. Smoking indoor could impact family members.			
10. Cigarette reduce reproductive functions.			

4.2 Attitude according to harms of smoking.

**ประเด็น**	**Agree**	**Disagree**	**Not sure**
1. Smoking is the way of relaxation.			
2. We can smoke in a public area.			
3. Smoking is terrible.			
4. Drinking alcohol must smoke			
5. If you have a lung problem, you should stop smoking.			
6. Smoke is not harmful for other people.			
7. Most components in cigarette are not harmful.			
8. Smoking can reduce weight.			
9. Cigarette is less harms than other substances.			
10. Male smoking is common.			

Part 5 Pulmonary capacities

Testing	Testing			Best test	Predicted value
	Trial 1	Trial 2	Trial 3		
FVC (L)					
FEV_1 (L)					
FEV_1/FVC (%)					
SPO_2 (%)					
